# Supplementary material for: The first dromaeosaurid (Dinosauria: Theropoda) from the Lower Cretaceous Bayan Gobi Formation of Nei Mongol, China
Source: PeerJ. 2015 Dec 7;3:e1480. doi: 10.7717/peerj.1480 (PMC4675113; doi:10.7717/peerj.1480)
Supplement: Supplemental Information 1 — A Chinese language abstract for interested locals from Nei Mongol and other parts of China. [file peerj-03-1480-s001.pdf]

Abstract for The first dromaeosaurid (Dinosauria: Theropoda) from the Lower Cretaceous Bayan Gobi Formation of Nei Mongol, China

内蒙古下白垩统巴音戈壁组的第一种驰龙科恐龙（恐龙总目：兽脚亚目）

摘要：本文报道了产于下白垩统巴音戈壁组的第一种驰龙科恐龙，其化石材料主要基于一件部分关连的不完整左侧后肢。这件标本的发现提升了这个地层的古生态系统中已知的生物多样性。这件后肢骨骼（IVPP V22530）具有典型的恐爪龙类特征，包括趾节 II-2 在膨大的近端和远端髁之间具有明显的收缩，以及趾节 II-3 显著增大。对称的脚掌以及细长的第五蹠骨表明这个个体属于驰龙类。尽管这件标本的准确分类位置还无法确定，另外两个特征表明其与于小盗龙亚科恐龙相类似。这些特征包括：第二、三、四蹠骨在远端紧密接合，以及趾节 II-2 内侧韧带窝腹缘靠近趾节 II-2 远端髁内侧中心。这种恐龙的分类位置需要更多标本的发现和研究来进行验证。无论 IVPP V22530 是属于小盗龙亚科还是其近亲，这件标本都对了解驰龙类的演化和生物学特征具有重要意义。IVPP V22530 还包括一个游离的指爪，一段右侧背肋的近端，以及一些包括肋骨在内无法鉴定的骨骼。其中部分散落的骨骼尚无法确定是否属于驰龙科恐龙，尽管它们和之前提到的左后肢有可能来自于同一个体。
